# Categories of important edges in dynamics on graphs

**DOI:** 10.1098/rsos.241086

**Published:** 2025-04-23

**Authors:** Dzmitry Rumiantsau, Johannes Falk, Piotr Nyczka, Marc-Thorsten Hütt

**Affiliations:** ^1^ Constructor University, Bremen, Germany; ^2^ Wrocław University of Science and Technology, Wroclaw, Poland

**Keywords:** dynamical edge importance, dynamical task, excitable dynamics, totalistic cellular automata, macaque cortical area network

## Abstract

How important is a single edge of a graph for a specific dynamical task? This question is of practical relevance to many research fields and is pivotal to understanding the structure–function relationships in complex networks more deeply. Here, we design an analysis strategy to answer it and explore the connection of such importance to network topology. Our approach for evaluating dynamical edge importance is based on the differences in time courses between dynamics on the original graph G and on the graph $G^{-}$ missing an edge. To demonstrate the method’s versatility, we apply it to two drastically different classes of dynamics—a minimal model of excitable dynamics, and totalistic cellular automata on graphs as representatives of pattern formation. Our results suggest that the dynamical usage of a graph relies on markedly different topological attributes for these two classes of processes. Finally, we study dynamical edge importance in the macaque cortical area network, to illustrate possible real-world applications. We find that dynamical importance of edges differ between the network and its switch-randomized counterparts, and these differences can be functionally interpreted. Moreover, they are qualitatively distinct for long-time courses and short transients, highlighting different parts of the network’s intended function.

## 1. Introduction

When a single edge is removed from a network, structural measures such as mean shortest path length, centrality or other graph invariants usually change [1]. By calculating the measures before and after the removal, the *edge importance*, that is, the influence of the edge on the corresponding measure, can be quickly determined [2].

An example is the induced centrality discussed in [3]. However, when considering dynamics on the networks instead of solely topological properties, the analysed systems become more involved [4,5]. Now, small changes, like the removal or rewiring of a single edge, can have significant and global effects that emerge over time through the intricate interplay between the graphs’ topology and the respective dynamics [6–10]. Calculating the *dynamical* edge importance, specific to a particular dynamics, is therefore challenging. Such a dynamical understanding of the importance of an edge is embedded in the general research field studying the relationship between the structure of a graph—the structural connectivity (SC)—and the dynamics running on it—the functional connectivity (FC) [11,12].

Strategies designed to assess the importance of edges for specific network functions exist in different disciplines under a variety of names and forms: immunization [13] or blocking [14] protocols for epidemic diseases or other types of infective spreading, edge importance in information transmission [15], edge ablation in large language models [16], the identification of critical edges in social network [17], edge importance assessed by incorporating transmission performance [18] or the impact of edge removal on the reproduction number in susceptible-infected-removed (SIR) dynamics [19]. Interactions of edge importance have been discussed in [20] in the context of spreading dynamics.

Here, we propose ways to calculate such *dynamical* edge importance. We focus on two fundamentally different dynamics, a cellular automaton (CA) with deterministic time evolution [21] and the stochastic susceptible-excited-refractory (SER) model [11] as a simple model of excitable dynamics. To obtain the *dynamical edge importance,* we define measures to quantify the difference between the dynamics run on the original graph and one in which an edge is missing. As soon as the dynamics contain probabilistic decisions, differences in the courses of the two dynamics cannot necessarily be attributed to the missing edge. For this reason, we develop a procedure to align the second simulation to the first one by matching the probabilistic decisions in a way compliant with the dynamical update rules. To obtain a statement on the relationship between SC and FC, we then compare the obtained dynamical edge importance with the graphs’ topological properties and calculate the correlations between both. Additionally, a cross-comparison between the edge importance of the two dynamics allows quantifying how the different dynamics require different topological properties.

As an illustration of possible applications of our method, we study edge importance in a real biological network, the macaque cortical area network [22]. By requiring links in both directions to be above the FLN threshold of 0.001 (FLN = fraction of labelled neurons), which encompasses strong and some of the pronounced moderate connections [23], we obtain an undirected, unweighted graph with 29 nodes and 89 edges. In this example, we compare the obtained dynamical edge importance with the ones obtained from switch-randomized counterparts of this network. We find that only in some cases the edge importance obtained from the real network are significantly different from the ones obtained from the randomized networks. For the CA dynamics, the observed differences can be attributed to the role of triangles. For the (in this example functionally more relevant) SER dynamics, these differences mostly occur in short transients, suggesting an optimization of the network with respect to this dynamical ‘usage’.

For some dynamics like the phase oscillators [24], there already exist, methods to link the performance of the dynamics (the synchronizability) to global topological measures, e.g. the eigenvalues of the Laplacian matrix [25]. For other dynamics like the SER model, it was shown that FC can be predicted by analysing small units of a graph, the pacemaker cycles [26]. However, dynamics on graphs serve as models in many more scientific fields, including biology [27,28], social sciences [29], logistics [30], operations research [31–33] and mathematics [34,35]. There, they provide valuable insight into neuronal activation [36], principles of pattern formation [21], disease or rumour spreading [37–39] and consensus finding [40,41]. Dynamical edge importance can be instrumental in gaining a more complete understanding of the correlation between FC and SC with respect to a wide range of dynamics. The methods we develop here are an important step in this direction. Using two generic and different models, we show how the edge importance can be calculated even if stochastic fluctuations make this difficult or only long-term effects, e.g. in the form of attractors, are relevant. We find that edge importance is not universal, but it is strongly process-dependent, as we see from the differences in edge importance and their structural characterization between the two dynamics. Furthermore, the details matter: in the same dynamics, transients can give a different pattern of edge importance than asymptotic behaviours.

The outline of the manuscript is as follows: in the next section, we first introduce the two dynamics. We then explain the procedure used to obtain the *dynamical edge importance* and subsequently explain how we align the stochastic runs to each other. In §3, we present our results for different stylized network topologies as well as for the macaque cortical area network. In the last section, we draw some conclusions and provide an outlook of where our results might have an impact.

## 2. Methods

In this study, we only consider simple graphs—unweighted, undirected graphs without self-loops or parallel edges. Throughout the investigation the *adjacency matrix* A of a simple graph G with nodes {1,2,…n} is defined as follows: $A_{ij}=A_{ji}=1$ if there is an edge between i and j in G , and $A_{ij}=A_{ji}=0$ otherwise.

The main quantity of our investigation is the *dynamical importance* of a given edge $e_{ij}$ . Informally, the process of computing this quantity can be described as follows. For a fixed discrete-time dynamical model and initial state $s_{0}$ , we run two copies of the dynamics starting from $s_{0}$ : on the original graph G and the graph $G_{e_{ij}}^{-}$ missing an edge. This results in time courses $T_{e_{ij}}$ and $T_{e_{ij}}^{-}$ , respectively. The dynamical importance of $e_{ij}$ , denoted by $\sigma_{s_{0}}(e_{ij})$ , is then defined based on normalized differences between specific subsets of those time courses, with the exact definition depending on the dynamical model and the type of network behaviour under investigation. The quantity $\sigma_{s_{0}}(e_{ij})$ is then averaged over a sufficient number of initial states. Repeating the process for all edges that do not fragment the graph when removed yields a distribution of $\sigma (e_{ij})$ , which can be used to identify edges with high and low dynamical importance.

**Figure 1.**
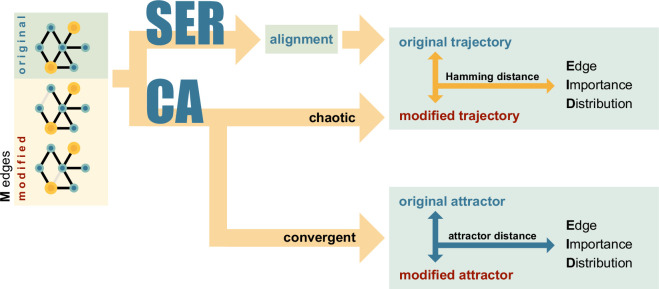
Outline of the experimental design. We start with our original network and a set of M modified networks. In each modified network another of the M possible edges is deleted. In our investigation, we analyse two different dynamical models, SER and CA. For the SER model, we compare the Hamming distance between the states of the model if running on the original network and the modified (edge deleted) network. For the CA model, we take two different approaches, depending on whether the model exhibits chaotic or convergent behaviour. To analyse the chaotic regime we compare—similar to the SER analysis—the Hamming distance between the dynamics running on the original and the modified network. To analyse the convergent regime, we compare the distance of the resulting attractors.

Though the process described above is fairly universal, precise computation of edge importance depends significantly on the chosen dynamical model. Therefore, the next part of this section is split between two subsections, each describing a single dynamical model and necessary adjustments for determining $\sigma (e_{ij})$ . A general summary of our investigation is shown in figure 1.

### 2.1. Edge importance in the SER model

The model assigns one of three states to each node—susceptible ( S ), excited ( E ) and refractory ( R )—which are updated synchronously in discrete time steps according to the following rules [11]:

(i) a susceptible node becomes an excited node, if there is at least one excitation in its direct neighbourhood. If not, it spontaneously becomes excited with probability f ;
(ii) an excited node enters the refractory state; and
(iii) a node in the refractory state becomes susceptible with probability p .

**Figure 2.**
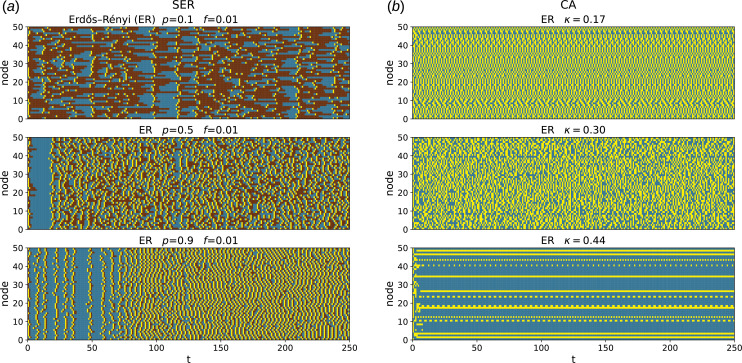
(*a*) Space–time plot of the SER dynamics on the Erdős–Rényi (ER) graph, with three different values of p . Colours correspond to these in figure 3. (*b*) Space–time plot of the CA dynamics on the ER graph, with three different values of κ . Transient included. Colours are: 0—blue and 1—yellow.

For the sake of convenience, we use the single-letter state names ( S , E and R , respectively). Figure 2*a* shows examples of space–time plots that result from running this dynamical model on the networks considered in our study.

**Figure 3.**
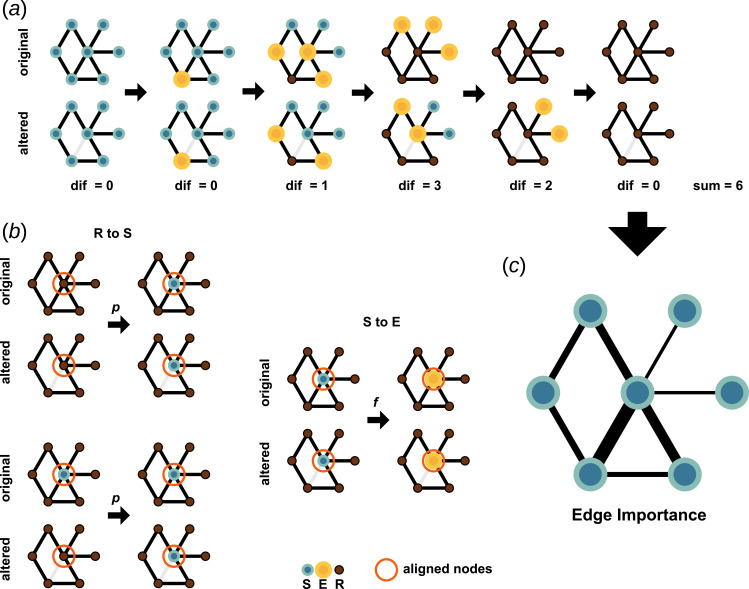
An illustration of edge importance computation for SER dynamics. (*a*) Short example of the dynamics run on the original and the altered (removal of a single edge) network, along with the calculated difference. Nodes in susceptible, excited and refractory states are coloured in blue-green, yellow and brown, respectively. (*b*) Alignment of stochastic events (change in the state of the node encircled in red in the original and modified runs). (*c*) Illustrates the edge importance and how it is derived.

A detailed description of the method used in this study to compute edge importance for SER dynamics is given in the appendix. It follows the description given at the beginning of §2, with two special considerations owing to the stochastic nature of the model. Figure 3 illustrates the process.

First, we perform random event alignment between original and modified dynamical runs. This is necessary to investigate the long-term effects of edge removal since omitting this step makes differences between said runs overshadowed by noise outside of very short time courses (electronic supplementary material, figure S7). There are two types of random events in SER dynamics: node recovery and excitation of nodes that have no excited neighbours. Before running SER dynamics on the modified network, outcomes of random events in the original dynamical run are recorded. This information is then used during the modified dynamical run to make it as close to the original as possible while observing the rules of SER dynamics. Denote by $S_{t}[b],S_{t}^{e}[b]$ the state of node b at time step t in the original and modified dynamical runs, respectively. Then $S_{t+1}^{e}[b]$ is determined as follows:

(i) if $S_{t}^{e}[b]=R$ and $S_{t}[b]=R$ , then $S_{t+1}^{e}[b]=S_{t+1}[b]$ ;
(ii) if $S_{t}^{e}[b]=S$ , $S_{t}[b]=S$ , and b has no excited neighbours at step t in both original and modified runs, then $S_{t+1}^{e}[b]=S_{t+1}[b]$ ; and
(iii) in all other cases, $S_{t+1}^{e}[b]$ is determined according to the update rules of SER dynamics.

Second, while random event alignment reduces the stochasticity of the modified run, it cannot completely eliminate it. Therefore, for every choice of edge and initial state, we perform k independent modified dynamical runs and compute the Hamming distance between each of k resulting time courses and the one from the original run. This list of distances is then averaged over k and the resulting mean is normalized by the number of time steps considered. The procedure is repeated for a sufficiently large set of initial states, and averaging the results over this set yields the importance of a given edge with respect to SER dynamics.

The resulting edge importance lies in [0,1] and represents the average fraction of nodes at every time step that has different states between modified and original runs.

Even though we are mainly interested in the long-term, far-reaching effects that alteration of a single edge has on the network, our method can be adapted to operate without one or both of those conditions. This is achieved by allowing the transient and/or the region around the affected edge to enter the calculations. In addition, by applying our method to the transient only, we gain insight into the immediate effects of single-edge alteration. We define the region around the affected edge as the union of neighbourhoods of vertices incident to said edge. Here is the list of both combinations of choices that we consider in this paper:

(i) excluding both the transient and the region. This is the default case, described above; and
(ii) considering only the transient and including the region.

We have also explored other combinations of including/excluding the transient and the region around the affected edge. However, the dynamical importance of those cases correlates strongly with the above two choices (electronic supplementary material, figure S1), so they do not add any qualitative differences to our investigation.

### 2.2. Edge importance for totalistic cellular automata

Apart from SER dynamics, we also adapt our method of computing edge importance for cellular automata. The model discussed in this study is a binary cellular automaton. Every node i∈{1,2,…n} in the network G has two possible states, $x_{i}=1$ and $x_{i}=0$ . At every time step t , future node states $x_{i}(t+1)$ are determined from present states $x_{i}(t)$ via the following update rule [21]:


$$\begin{array}{c} \begin{array}{r} x_{i}(t+1)=\left\{ \begin{array}{cc} x_{i}(t), & \rho_{i}\leq \kappa \\ 1-x_{i}(t), & \rho_{i}>\kappa , \end{array}\right. \end{array} \end{array}$$

where $\rho_{i}$ is the excitation density in the neighbourhood of node i at time step t and κ is the model parameter. Throughout the paper, this model will be referred to as CA dynamics. Figure 2*b* shows examples of space–time plots that result from running this dynamical model on the networks considered in our study.

Depending on κ , the system is either convergent or chaotic (convergence is not reached within the iteration limit; electronic supplementary material, figure S8). Since our primary interest lies in the long-term effects that removing a single edge has on the system, we found it more meaningful to compute edge importance based on the steady states of the original and modified runs in case of convergence. Therefore, we employ two different variations of our method depending on the dynamical regime, which is described in detail in appendix A.

For chaotic CA, edge importance is computed very similarly to the process described at the beginning of §2. Averaging happens only over the set of initial states. The resulting edge importance lies in [0,1] and represents the average fraction of nodes at every time step that has different states between modified and original runs. We base the edge importance on the transient part of the dynamics because the distribution of long-term-based edge importance in that region of κ did not have a meaningful width (electronic supplementary material, figure S2). The region around the affected edge is included in the calculations.

For convergent CA, the procedure is significantly different. For a fixed edge, both original and modified dynamical runs are iterated until convergence, and the importance of an edge is computed based on the distance between the two attractors. Then, it is averaged over a sufficiently large number of initial states.

The idea behind the attractor distance we employ is to measure how well the longer attractor can be split into consecutive blocks, each of which acts like the shorter one. Its choice was motivated by the following considerations:

(i) it should return a Hamming distance for fixed-point attractors;
(ii) it should return 0 for two different circular shifts of the same cyclic attractor; and
(iii) it should not inflate the importance of edges just because they happen to converge to cyclic attractors, that is, the average distance between two randomized versions of an attractor should be the same, regardless of length.

### 2.3. Numerical experiments

In this study, we consider two sets of synthetic networks: 120 ER networks of 50 nodes with 100 links, and 120 Barabási-Albert (BA) networks of 50 nodes with m=2 . They remain the same for all modelling choices and dynamical parameters and will be referred to as *ER network set* and *BA network set* for the sake of convenience

#### 2.3.1. SER parameters

We consider the following two set‐ups:

(i) both the transient and the region around the affected edge are excluded from the calculation. The dynamics are iterated for N=500 time steps, and the first $N_{0}=100$ are not considered. Edge importance is averaged over a set M of five random initial states, each component is chosen uniformly at random between S , E and R . In this instance, statistical information is obtained from several long-time courses, rather than a large number of short transients. Importance values computed in such a fashion reflect long-term changes shaped by a stochastic dynamical process in conjunction with network structure. Hence, they should not strongly depend on the network initialization. In fact, we found that |M|=5 is sufficient to yield reliable results over the course of repeated simulations; and
(ii) only the transient is considered, and the region around the affected edge is included. The dynamics are iterated for 50 time steps. Edge importance is averaged over a set M of 100 random initial states, each component is chosen uniformly at random between S and E .

Regardless of other parameters, k=5 modified dynamical runs are performed for each choice of edge and initial state to account for the stochastic nature of the SER model.

The SER dynamics is run for p∈{0.1,0.3,0.5,0.7,0.9} and f=0.01 .

#### 2.3.2. Cellular automation parameters

(i) *Convergent CA*. The dynamics are iterated until convergence. Edge importance is averaged over a set M of 1000 random initial states. For the ER network set, we experimentally identified two regions of convergence: one for low κ , which corresponds to comparatively long cyclic attractors, and one for high κ , where we observe short cycles and fixed point attractors. We investigate κ∈0.11,0.13,…,0.19 and κ∈0.36,0.38,0.40,…0.50 , for both ER and BA network sets.
(ii) *Chaotic CA*. Dynamics are iterated for N=50 steps, and the region around the affected edge is included in the calculation. Edge importance is averaged over a set M of 100 random initial states. We investigate κ∈0.26,0.28,0.30,0.32 .

#### 2.3.3. Topological properties of edges

Several topological quantities were used for studying the dependence of the edge importance on the network structure. The list of such quantities is given below:

(i) *incident degree centrality (IDC)*: average degree centrality of edge endpoints;
(ii) *incident eigenvector centrality (IEC)*: average eigenvector centrality [42] of edge endpoints;
(iii) *betweenness centrality (BC)* [43];
(iv) *hub-set-orientation prevalence (HSO)*: a measure based on shortest paths that calculates the orientation of an edge with respect to a set of hubs by fixing an enumeration of edge endpoints and counting which of them is more often reached first from the hub set by following the shortest path [44]. In this study, we use the whole network as the set of hubs;
(v) *incident degree asymmetry (IDA)*: the absolute value of asymmetry [45] of edge endpoint degrees;
(vi) *topological overlap of an edge (TO)*: fraction of network nodes that are connected to both endpoints of said edge; and
(vii) *incident clustering coefficient (ICC)*: average clustering coefficient [43] of edge endpoints.

### 2.4. Why do we use these network models?

Before applying our methods to real-world networks, we wanted to develop them using synthetic data. ER and BA graphs are two well-studied random graph models that are commonly used in network science. In the context of our study, they also illustrate the effect that network architecture has on the distribution of dynamical edge importance (given the same network size and similar connectivity). To investigate the applicability of our approach to real-world networks, we augment this exploration by using a network of the macaque cortical area. In the future, it would be interesting to consider other random network architectures (such as the Watts–Strogatz model), as well as real-world networks from different domains, but those experiments lie beyond the scope of this study.

### 2.5. Why were those two dynamical models selected?

The two models discussed in our study were chosen for several reasons. Both of them have only a few parameters, which allows for an exhaustive analysis. The SER model is a representative of the large class of excitable dynamics, which have numerous real-world applications, in particular in neuroscience [11,44]. Totalistic cellular automata serve as an example of dynamics capable of pattern formation. This phenomenon has been of interest to network science and computational biology, so we wished to establish a foundation for considering dynamical edge importance in that context.

In addition, the two dynamics are markedly different from a technical perspective: one is stochastic, the other deterministic and the latter exhibits qualitatively different steady-state sets across the parameter space. Not only did those distinctions between the models enhance our understanding of dynamical edge importance, but they were also very helpful for developing strategies that would generalize our approach to other kinds of dynamics.

Finally, although neither chosen model has direct real-world applications, the fact that they allow our method to be employed successfully implies a similar possibility for other, more complex models from the same dynamical class. Naturally, model-specific adjustments will be required, but a discussion of the general guidelines for doing so is provided in the next section.

### 2.6. How can this method be applied to other dynamics?

Even though the exact algorithm for computing edge importance strongly depends on the chosen dynamical model, we can provide guidelines for applying our method to other models based on examples considered in our study. Two main factors determine the correct approach: convergence of dynamics to a steady state, and presence of stochasticity in dynamical update rules.

Dynamical convergence suggests whether the calculation of edge importance should be based on some measure of difference between steady states in the original and modified runs, or on the difference between time courses in said runs. It is, of course, technically possible to use time courses for defining edge importance even in convergent dynamics, but in such a case we leave the choice of the more meaningful definition of the two up to the reader. Adhering to those guidelines may sometimes result in different procedures for computing the dynamical edge importance across different regions of the parameter space for the same model (see §2.2 for an example).

In case the update rules of the model are stochastic, it may be necessary to implement random event alignment between original and modified runs. Details depend on the model in question, but the main idea of alignment is to record stochastic decisions that are made during the original run, then make sure that at every time step of the modified run those decisions happen in the same way (as much as possible without violating the rules of the model). An example of such a procedure is described in § 2.1. For the benefits of adopting this approach, see §4.

With that in mind, we can sketch a schematic description of the algorithm for computing edge importance in a simple graph G over a set of initial states M :

(i) compute list of edges removing which keeps G connected, $L_{r}$

(ii) for each initial state s∈M , first run the dynamical model on G starting from s , obtaining either a time course $T_{s}$ or a steady state $a_{s}$ (depending on model convergence, see step 2.1 in appendix A(ii) and A(iii), respectively);
(iii) then, for each edge $e\in L_{r}$ consider G with e removed (denoted by $G_{e}$ ) and run dynamics starting from s on $G_{e}$ . If the model is stochastic, perform random event alignment while doing so. Results of this step are either a single time course $T_{s}^{e}$ (steady state $a_{s}^{e}$ ), or a set of such quantities obtained from multiple runs starting at s on $G_{e}$ (in case randomness could not be eliminated completely, see step 2.2.2 in appendix A (i) for an example);
(iv) now, compute $\sigma_{s}(e)$ —dynamical importance of edge e with respect to state s . It is defined either as the sum of distances between $T_{s}$ and $T_{s}^{e}$ at each time step (normalized by number of nodes and time course length, see steps 2.2.3−2.2.4 in appendix A(ii)), or as some measure of difference between $a_{s}$ and $a_{s}^{e}$ (normalized by the number of nodes see steps 2.2.3−2.2.4 in appendix A(iii)). In case a set of quantities was obtained on the previous step, differences between original run and every element of this set are computed, then averaged (see steps 2.2.3−2.2.4 in appendix A(i)). The choice of distance measure depends primarily on the state space of the model in question; and
(v) finally, average $\sigma_{s}(e)$ over all states s∈M to obtain σ(e) —dynamical importance of edge e . A distribution of σ(e) over all edges in $L_{r}$ is the final output of our method.

## 3. Results

To better understand the phenomenon of dynamical edge importance, as well as the extent of its dependence on modelling and dynamical choices, our exploration happens on several levels: (i) using two sets of synthetic random networks with different architectures, we study both long-term and transient-based variations of edge importance with respect to stochastic dynamical model (SER) for a broad range of recovery probabilities, and the connections of this quantity to various topological properties of the underlying network. (ii) on the same set of networks, we study dynamical edge importance with respect to totalistic cellular automata for wide ranges of κ in both chaotic and convergent dynamical regimes; (iii) we investigate the connections between the different edge importance that result from our modelling and dynamical choices; and (iv) Using a network of the macaque cortex, we assess the applicability of our methods to biological networks.

### 3.1. SER edge importance

**Figure 4.**
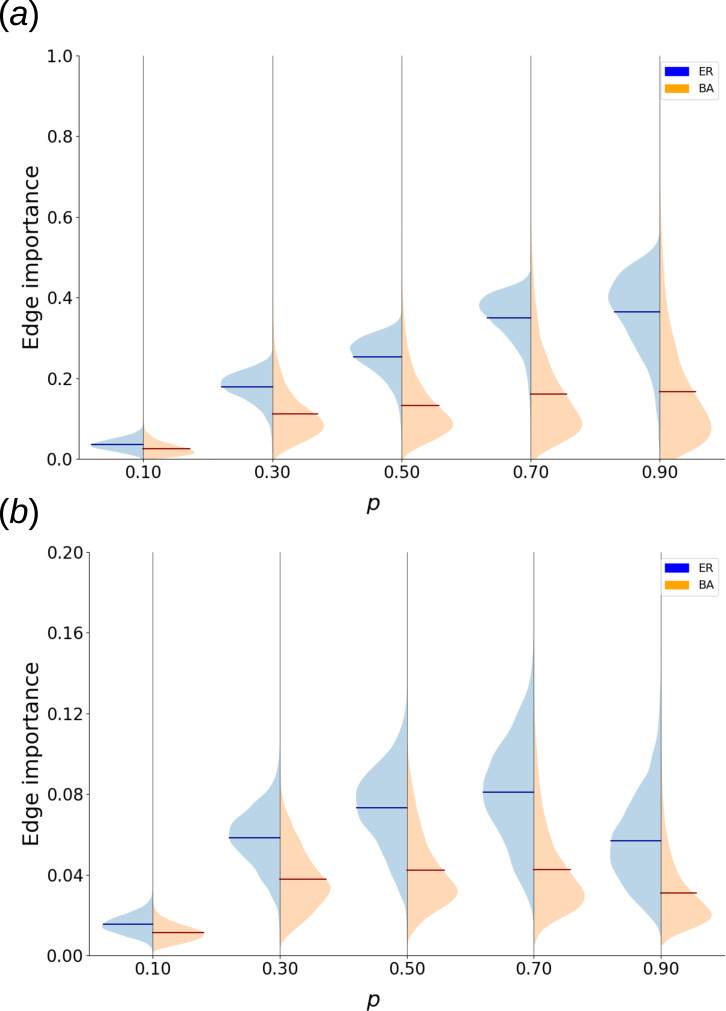
(*a*) Violin plots of collective SER edge importance distributions for 120 ER (blue) and 120 BA (orange) graphs depending on recovery probability. Time series of 500 steps were used, excluding 100 steps of the transient and the region around the deleted edge from the calculation (§2). (*b*) Violin plots of collective SER edge importance distributions for the same sets of ER and BA graphs as in A. Short time series of 50 steps were used, and the region around the deleted edge was included in the calculation (§2).

Applying our methods to the SER model allows us to understand their robustness and relevance in a stochastic setting and illustrates the significance of dynamical edge importance in excitable processes. Figure 4 shows the dependence of SER dynamical edge importance on recovery probability for both ER and BA network sets (§2).

Based on it, several important observations can be made. The quantity exhibits a meaningfully broad distribution, enabling the study of edges that assume extreme values of importance. However, network architecture has a strong impact on the shape of edge importance distribution, suggesting a significant topological connection. Likewise, recovery probability significantly affects the range of values that enter the distribution, in a non-monotonous fashion.

Our findings demonstrate that the removal of a single edge, which is a minor structural change, can have a drastic long-term impact on SER dynamics that is captured by the notion of dynamical edge importance. To no surprise, however, the extent of this effect strongly depends on network architecture, with the average fraction of nodes different at every time step between dynamics on original and modified networks being above 30% in some regions of parameter space for the ER network set, but never rising above 20% for BA graphs.

It is also interesting to consider SER edge importance based on long-term behaviour (figure 4*a*) alongside the version based on transients (figure 4*b*). The values of those quantities for corresponding recovery probabilities differ by almost an order of magnitude, demonstrating a nonlinear propagation of state differences caused by edge removal in the network. Curiously, the shapes of corresponding importance distributions differ significantly for the ER network set but are similar for the BA network set. Basing edge importance on the transient of a time series seems to create a noticeable fraction of highly important and sensitive edges in ER networks: on average, 2.5% of edges have a transient importance more than 2 s.d. above ER mean (as opposed to 0.7% for long-term importance).

**Figure 5.**
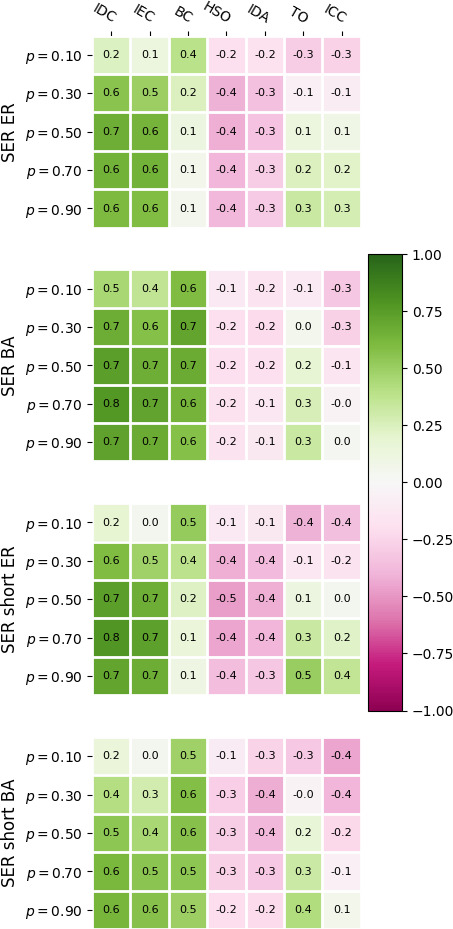
Heat map of average Pearson correlations between SER edge importance of all valid edges in a network and topological properties of those edges, averaged over the same sets of 120 ER and 120 BA networks for each choice of dynamical parameters. For the exact definitions of said topological properties, see §2.

To investigate connections between edge importance and topological properties of a network, we compute the Pearson correlations between edge importance and specific edge-based network properties (§2), and average the results over ER and BA network sets (figure 5). For low recovery probability (*p* = 0.1), the importance based on long-time courses in the ER set is connected to the betweenness centrality (correlation of 0.4), but otherwise relatively decoupled from topology. As p increases, this connection disappears and instead gives rise to stronger coupling with the incident degree and the eigenvector centralities (correlation greater than 0.6). A possible explanation for this connection is that high recovery probability enhances the frequency of hubs spreading the state differences caused by removing a link between them to the remainder of the network. This remains true for transient-based importance in the same set with one significant difference: in this case, the connection to betweenness centrality remains strong across the whole range of p (minimum observed correlation is 0.5), suggesting that the aforementioned fraction of highly sensitive edges consists chiefly of those with high values of both incident degree centrality and betweenness centrality. As expected, the BA network set exhibits stronger ties to topology and shows no qualitative differences between long- and short-term-based versions of edge importance. Overall, our results suggest a strong link between dynamical edge importance in the SER model and degree-based centrality measures for high recovery probabilities and a somewhat weaker link to path-based centrality measures for low recovery probabilities. Given the excitable nature of dynamics, this is not surprising. However, the nature of the link between dynamical importance and topology seems to depend significantly on the interplay between network architecture and the length of dynamical trajectories used in the definition (which reflects the type of dynamical network response that we wish to analyse).

### 3.2. Cellualr automation edge importance

**Figure 6.**
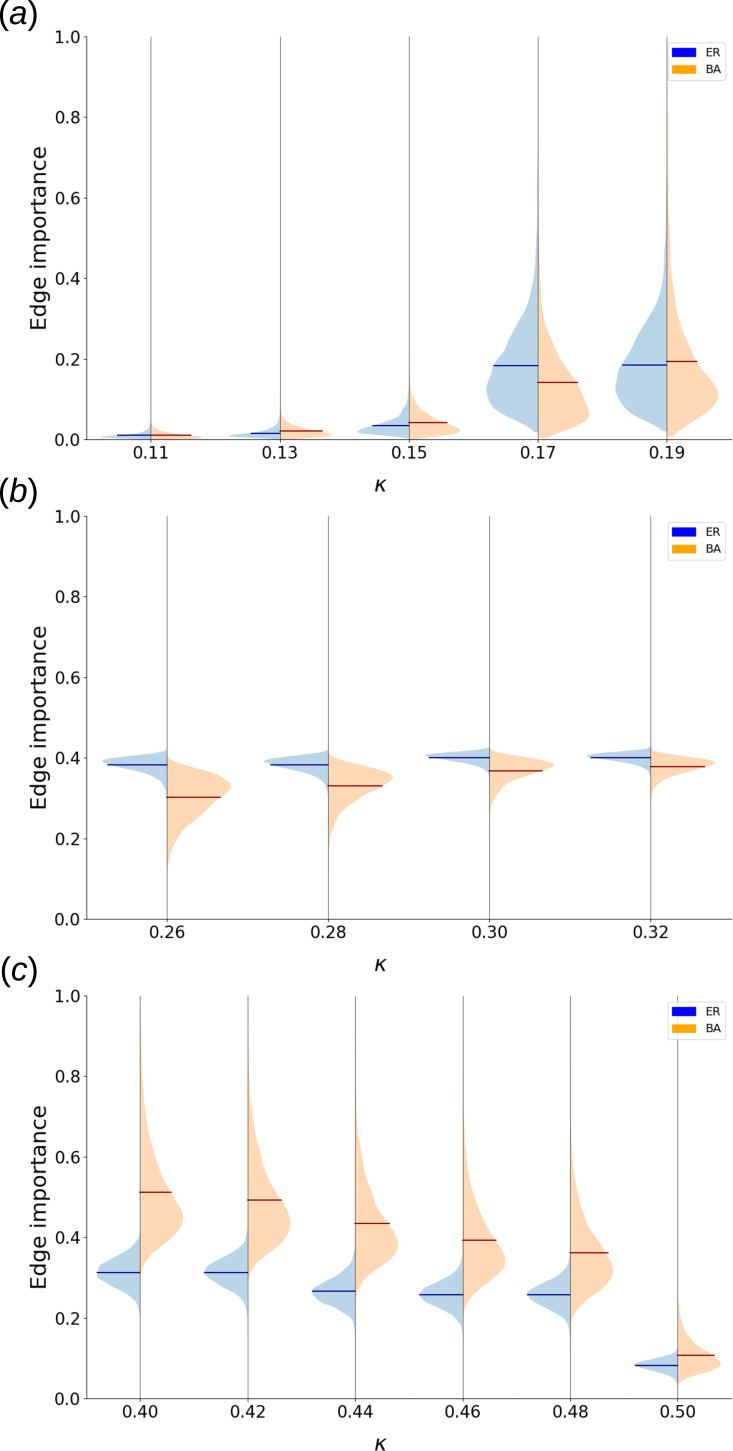
(*a*) Violin plots of collective convergent CA edge importance distributions (§2) for 120 ER (blue) and 120 BA (orange) graphs in low κ range. The region around the deleted edge was excluded from the calculation. (*b*) Violin plots of collective chaotic CA edge importance distributions for the same sets of ER and BA graphs as in (*a*). Short time series of 50 steps were used, and the region around deleted edges was included in the calculation (§2). (*c*) Violin plots of collective convergent CA edge importance distributions for the same sets of ER and BA graphs as in (*a*) and high range of κ . The region around the deleted edge was excluded from the calculation.

Being a deterministic minimal dynamical model capable of pattern formation, totalistic cellular automata serve as a very informative complement to SER dynamics when studying edge importance. Figure 6 shows the dependence of edge importance for convergent and chaotic CA on κ for both ER and BA network sets (§2).

For the ER network set, we experimentally identified two regions of convergence: one for low κ (figure 6*a*), which corresponds to comparatively long cyclic attractors, and one for high κ (figure 6*c*), where we observe short cycles and fixed point attractors.

In the low κ region, edge importance distributions exist on widely different scales, despite seemingly minute changes in dynamical parameters (average ER edge importance is 0.03 for κ=0.15 , and is 0.18 for κ=0.17 ). Two points are of particular note: the pronounced long tail of the distribution for ER network set at κ=0.17,0.19 , and the similarity between ER and BA network sets in this region.

In the high κ region, the BA distributions retain the long-tailed shape they have shown before, while ER distributions become noticeably more symmetric. The value ranges between the two network sets are strikingly separate (as an example, with ER edge importance in (0.16,0.42) and BA edge importance in (0.29,1.63) for κ=0.4 ) for 0.4<κ<0.5 , but grow closer afterwards.

Figure 6*b* shows the transient-based edge importance for the chaotic CA region. While the importance values themselves are prominent (40% affected nodes on average at every time step) neither network set exhibits a sizable fraction of particularly important edges.

At low κ , the jumps of edge importance distribution scales are owing to the interplay between the dynamical update rule and degree distribution of the underlying networks. ER networks in our study exhibit node degrees of up to 13, and BA networks of up to 30. Changing κ from 0.11 to 0.13 only alters the dynamical behaviour of nodes with degrees 8, 9, 16−18 and 24−27; the same holds for the change from 0.13 to 0.15 and degrees 7, 14, 15, 21−23, 27−30, as well as the change from 0.15 to 0.17 and degrees 6, 12, 13, 18, 19, 24−26, 30. Corresponding fractions of affected nodes are approximately 3%, 6% and 11% in ER networks, and approximately 5%, 6% and 7% in BA networks we analyse (electronic supplementary material, figure S9). This accounts for the aforementioned jumps in the ranges of edge importance distributions. Although the disparity between fractions of affected nodes themselves is not as drastic in BA networks, with increasing κ hubs start to exhibit an increasing variety of dynamical behaviour, so increasing the range of the edge importance distribution is natural if we consider its growing connection to the betweenness centrality in that region of κ .

The effects of degree distributions on the range edge importance become more pronounced for high values of κ . For instance, increasing κ from 0.44 to 0.48 only changes the dynamical behaviour of nodes with a degree of at least 9, which make up less than 2% of all nodes in ER networks (electronic supplementary material, figure S9). By contrast, BA networks have significantly broader degree distributions, and nodes of degree of at least 9 not only comprise approximately 8% of the network but also have stronger ties to edge importance as evidenced by its connection with incident degree centrality and betweenness centrality. This explains the difference between the edge importance distributions that we observe for ER and BA networks.

**Figure 7.**
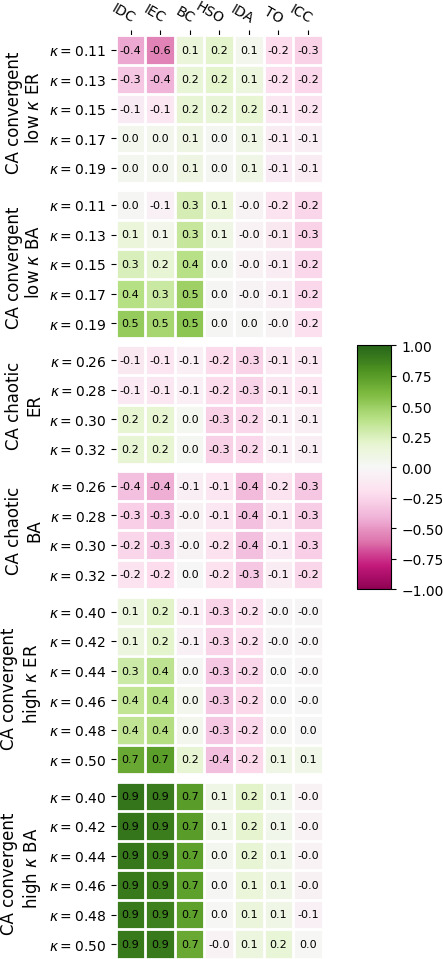
Heat map of average Pearson correlations between CA edge importance of all valid edges in a network and topological properties of those edges, averaged over the same sets of 120 ER and 120 BA networks for each choice of dynamical parameters. For the exact definitions of said topological properties, see §2.

Figure 7 shows correlations between CA dynamical importance and topological network properties (§2). Apart from extreme values of κ , convergent CA edge importance in the ER network set does not seem to have significant ties to topology. The same cannot be said for the BA network set: it correlates with betweenness centrality that lies in the range [0.3,0.5] in the bulk of low κ region, has a consistent correlation of approximately 0.9 with incident degree centrality throughout the high κ region. The latter phenomenon probably occurs because the removal of a link between two hubs makes the state of those hubs change one step sooner than in the original run, and those differences propagate to a significant fraction of nodes in the network owing to specifics of BA architecture, which results in a substantially different attractor. It also has a noticeable ( −0.375 on average across all relevant values of κ ) negative correlation with incident in-degree asymmetry in the chaotic κ region, though the ER network set shares this feature to some extent (with an average correlation of −0.25 ).

Summarizing, our findings suggest that although the range of edge importance based on totalistic cellular automata is strongly influenced by network architecture and degree distribution, the importance of individual edges is reasonably decoupled from network topology outside of the high κ region. Since the minimal model was chosen as a representative of pattern formation, a weaker link to topology is in line with the model’s character. This result also makes it an example of a system where a small structural change (edge removal) leads to significant dynamical changes that cannot easily be traced back to network structure and explained from that perspective.

While comparing those results to our findings for SER dynamics, two interesting observations can be made. First, the shapes of edge importance distributions in BA networks are qualitatively similar between SER and CA models in certain parameter regions ( κ=0.17,0.19 and κ≥0.4 , see figures 4 and 6). Given that figures 5 and 7 show similar topological connections to edge importance in those cases, it suggests that certain network architectures can be somewhat robust in terms of dynamical importance even under a drastic change of dynamical model, with BA graphs being one example. Our method can help identify such architectures, which seem better suited for executing a multitude of markedly different dynamical tasks and may therefore be of interest to research that happens on the intersection of several application domains. Second, the impact of network architecture on edge importance varies qualitatively across CA parameter space, unlike the SER model. Whether this is a result of a qualitative difference between the parameter regions, or a distinct character of dynamical models, such variation suggests that dynamical edge importance depends on the interplay between structural and dynamical factors, rather than simply the factors themselves.

### 3.3. Correlations between edge importance for different dynamics and modelling choices

**Figure 8.**
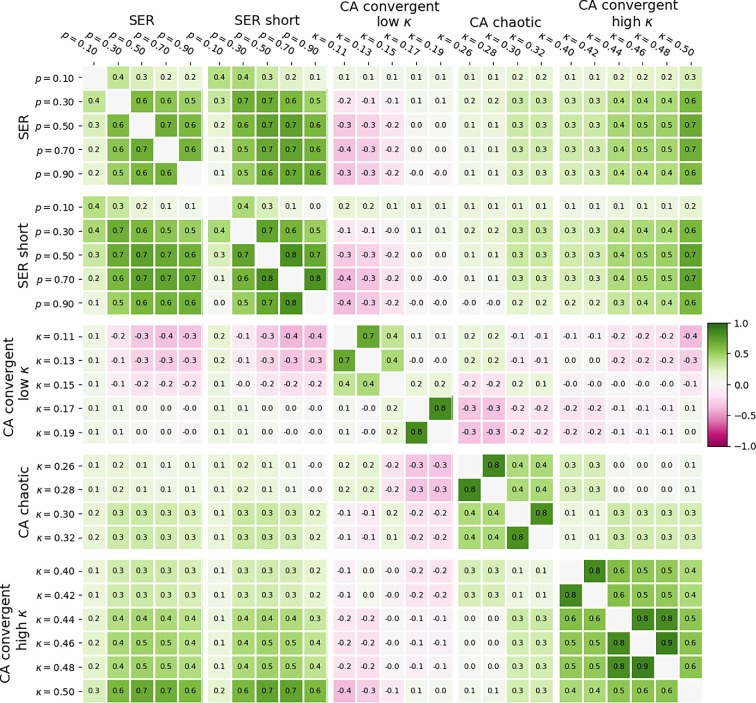
Heat map of Pearson correlations between edge importance for different dynamic and modelling choices (§2). The same set of 120 ER networks was used in every instance.

It is only natural to investigate how various versions of edge importance studied in this article relate to one another. Figure 8 features correlations between all edge importance versions on synthetic networks that we considered for the ER network set (see the electronic supplementary material, figure S3, for a similar heatmap of the BA network set).

Based on that figure, we can draw the following conclusions:

(i) SER edge importance based on long-time courses has strong correlations (greater than 0.5 for the same value of recovery probability) with transient-based SER edge importance;
(ii) transient-based chaotic CA edge importance tends to have weak negative correlations with convergent CA edge importance in the low κ region (−0.25 on average for κ=0.17,0.19 ) and weak positive correlations with the same quantity in the high κ region (0.1625 on average of κ>0.42 ). Convergent CA edge importance in low and high κ regions have weak negative correlations (approximately − 0.1 on average across all considered values of kappa); and
(iii) for 0.17≤κ≤0.28 and any values of p , there seems to be no significant linear dependence between the SER edge importance and the CA edge importance. This is also true for p≤1 and any values of κ . However, SER edge importance exhibits noticeable negative correlations with convergent CA edge importance for low κ (−0.25 on average for p≥0.3 and κ≤0.15 ), and strong positive correlations with the same quantity for high κ (approximately 0.49 on average for p≥0.3 and κ≥0.44 ). Especially interesting is the case of high p≥0.3 and κ=0.5 , where the edge importance for SER and CA are not only highly correlated between themselves, but both are also strongly tied via correlation to IDC and IEC.

Overall, those results illustrate how dynamical edge importance changes across the combined landscape of dynamical, parametric and modelling choices, with the following key observations. Although the importance based on long-time courses and short transients highlights different aspects of networks’ dynamical function, there is a rather strong link between those two quantities in SER dynamics. Importance values obtained from qualitatively different regions of CA parameter space are mostly decoupled from one another. The link between SER and CA importance is positive in parameter regions where those models have the same strong topological drivers, and negative where they strongly correlate with those drivers in opposite ways. However, in regions where either of the two importance values has no strong connections to topology, the two definitions appear to be independent. Such regions are of particular interest if one wishes to compare how networks perform with respect to markedly different dynamical tasks while also minimizing the impact of network structure.

### 3.4. Macaque cortical area network results

**Figure 9.**
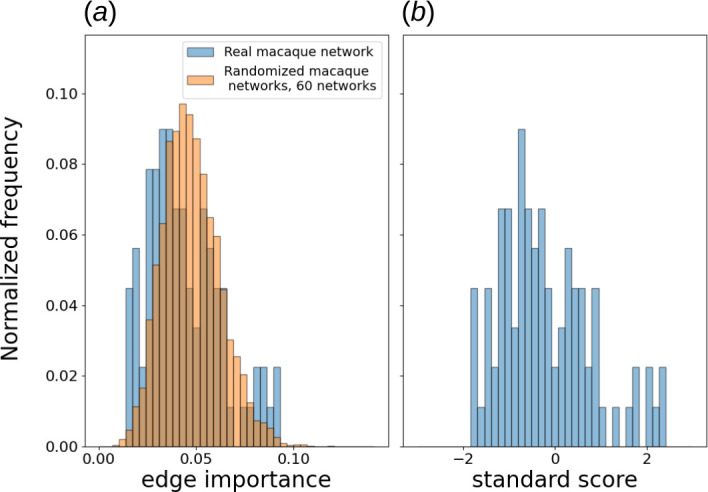
(*a*) SER edge importance distribution of actual macaque cortical area network versus collective distribution of its 60 switch-randomized instances for p=0.5 . Five hunderes time steps, 100 steps transient and the region around the deleted edge were excluded from the calculation. (*b*) Distribution of corresponding standard scores of macaque cortical area network edge importance with respect to randomized importance distribution.

**Figure 10.**
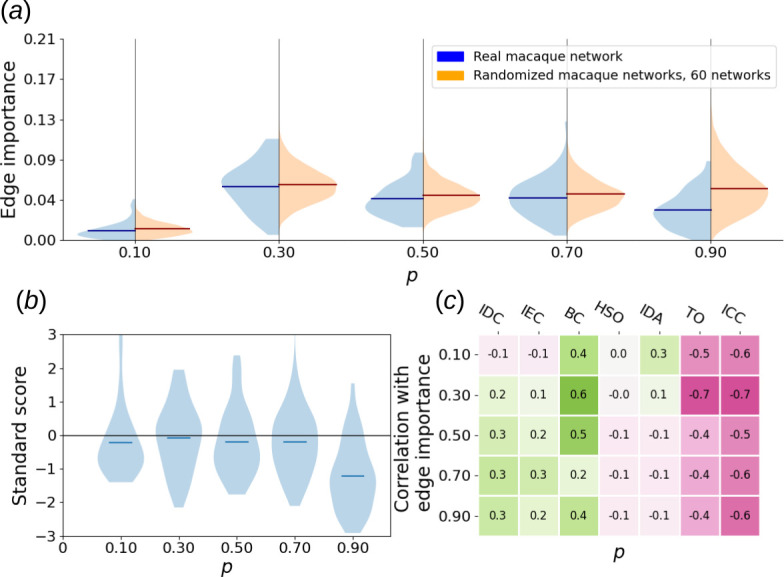
(*a*) Violin plots of SER edge importance distribution of actual macaque cortical area network versus collective distribution of its 60 switch-randomized instances. Five hundered time steps, 100 steps transient and the region around the deleted edge were excluded from the calculation. (*b*) Violin plots of corresponding standard scores of macaque cortical area network edge importance with respect to randomized importance distribution. (*c*) Heat map of Pearson correlations between SER edge importance of all valid edges in macaque cortical area network and topological properties of those edges. For the exact definitions of said topological properties, see §2.

**Figure 11.**
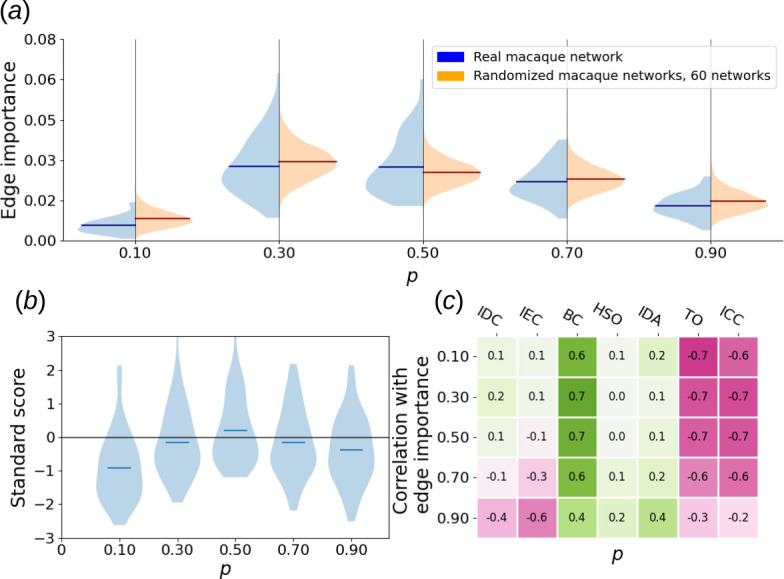
(*a*) Violin plots of SER edge importance distribution of actual macaque cortical area network versus collective distribution of its 60 switch-randomized instances. Fifty time steps, the region around the deleted edge was included in the calculation. (*b*) Violin plots of corresponding standard scores of macaque cortical area network edge importance with respect to randomized importance distribution. (*c*) Heat map of Pearson correlations between SER edge importance of all valid edges in the macaque cortical area network and topological properties of those edges. For the exact definitions of said topological properties, see §2.

To study the applicability of our methods for biological networks, we have taken the network from [22] and obtained its binary symmetric adjacency matrix by requiring both i→j and j→i to be above the FLN threshold of 0.001 , which encompasses strong and some of the pronounced moderate connections [23], resulting in an undirected, unweighted graph with 29 nodes and 89 edges. We calculated all five types of dynamical edge importance defined in this article across all considered values of dynamical parameters. To assess statistical significance, we repeated this procedure for 60 switch-randomized network versions and obtained collective edge importance distributions. Figure 9 shows an example of such distributions for a single parameter choice, while figures 10 and 11 offer a broader scope.

Based on those experiments, edges in the macaque cortical area network respond differently to long- and short-term excitable dynamics. When SER importance is based on long-time courses (§2), the distribution of edge importance is quite similar to that of randomized graphs, perhaps with a more pronounced group of edges with very low edge importance present in the real network (figure 10). A stronger bias towards low-edge importance can be seen as a higher overall robustness of the network with respect to long-term dynamics.

**Figure 12.**
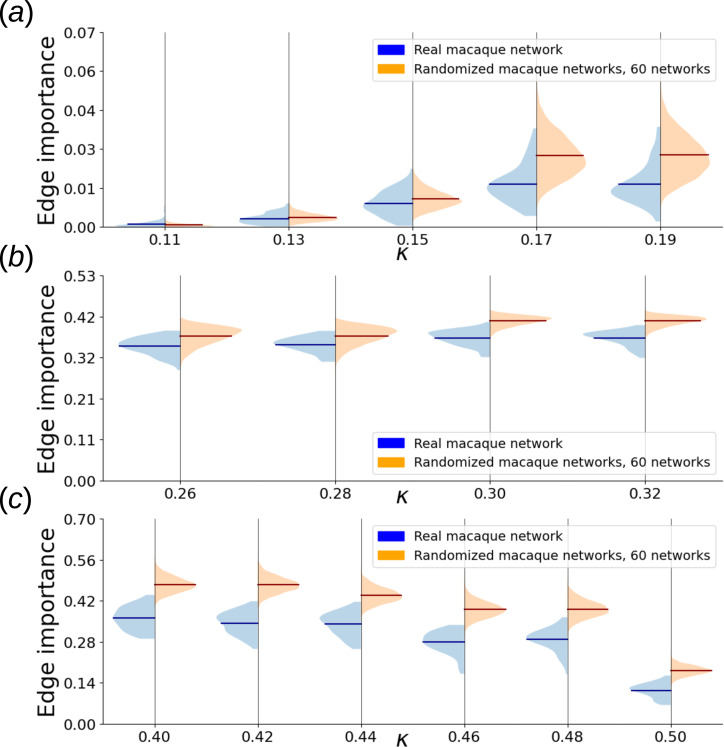
Violin plots of CA edge importance distribution of actual macaque cortical area network versus collective distribution of its 60 switch-randomized instances (this set is the same in all three subfigures). (*a*) Convergent CA, low κ range. Importance evaluated using attractor distance (§2). (*b*) Chaotic CA edge importance distributions. Short time series of 50 steps were used, and the region around the deleted edge was included in the calculation (§2). (*c*) Convergent CA, high κ range. Importance evaluated using attractor distance (§2).

However, for short transients, edges with much higher edge importance are found in the real network, compared to its switch-randomized counterparts (figure 12). This set does not exist for longer time series and suggests a tendency towards sensitivity with respect to external stimuli.

Our statistical analysis confirms those observations. For all values of recovery probability, differences between real and switch randomized distribution are statistically significant with a *p*-value under 0.05 according to a two-sample Kolmogorov–Smirnov test. Additionally, for all recovery probability values except p=0.3 and p=0.7 , those differences are significant with a *p*-value under 0.01 according to the same test. For long-time courses, the fraction of edge importance at least 1 s.d. above the mean is 0.153 in the real macaque network, which is an 8.2% decrease compared to the same quantity in randomized graphs, calculated after removal of the outliers from the distribution (using the 1.5 interquartile range threshold). Excluding the value p=0.1 , the fraction of edges with importance at least 2 s.d. above the mean is on average 13.4% higher in the actual macaque network than in the randomized ones. For transients, however, the fraction of importances 1 s.d. above the mean is 8% higher in the real network compared to randomized. More importantly, edges with importance at least 2 s.d. above the mean become on average 44% more frequent in the real network, which substantiates our hypothesis about high sensitivity to external stimuli. A shift to transients also strongly enhances the statistical significance of distribution differences, with the highest observed *p*-value in the two-sample Kolmogorov–Smirnov test being $4.38\times 10^{-4}$ .

In both cases, edge importance in the macaque cortical area network has a noticeable positive correlation with betweenness centrality (0.42 on average for long-term and 0.6 for transient importance), and a negative correlation with topological overlap (−0.48 and − 0.6 on average for long- and short-term importance, respectively).

CA-based importance of edges in the macaque cortical area network is shifted towards lower values than that of networks’ randomized versions (see figure 12 and the electronic supplementary material, figures S4–S6, for *z*-scores and topological correlations). With increasing κ , the separation between real and random CA-based importance distributions becomes ever more pronounced. We assume that it is owing to the fact that switch randomization is not triangle preserving. The real macaque network has 88 triangles, compared to 38.05±3.98 in the set of switch-randomized graphs. So, the differences in edge importance distributions between the real network and the switch-randomized networks are indicative of the relevance of triangles in the CA dynamics.

In the case of convergent CA, the edge importance has a significant negative correlation with the incident clustering coefficient for both high and low κ regions (on average, − 0.48 for low and − 0.44 for high). However, this effect is also present for chaotic CA, and no topological quantity considered in this study satisfactorily explains it (see the electronic supplementary materials, figures S4–S6, for details).

The differences between SER and CA edge importance in the macaque cortical area network indicate the actual function of said network. Overall, SER dynamics, which are a realistic model for questions in neuroscience [11,44], produce significantly lower values of edge importance than CA, suggesting that the network indeed evolved for robust handling of excitable dynamics. In addition, values of κ=0.17,0.19 , which result in a shape and range of edge importance distribution that are closest to the SER model, also provide the same topological link to edge importance—betweenness centrality (electronic supplementary material, figure S4). This analysis demonstrates that our method can be applied to the macaque network, and yields biologically interpretable results when excitable dynamics are considered. Further insights into the function of this system can be obtained by using our approach to analyse a more specialized model on the same network, and interpreting the resulting edge importance distribution.

## 4. Discussion and outlook

With this investigation, we have made a step towards a better understanding of the relationship between FC and SC. We have investigated two types of dynamics on three network topologies: two stylized artificial models and one derived from biology. We focused on the local aspects of the problem at hand, using dynamical edge importance as our main quantifier. For the calculation of the edge importance, we compared the original trajectory of the system with the trajectory of the modified system where the singular edge in question was deleted. For both systems we always started from the same initial conditions and aligned random events when necessary, to keep the trajectories as close as possible. This technique helped us to expose edge importance.

Finally, we compared the obtained edge importance with a few classical topological quantifiers of the network edges. Our approach exposed substantial qualitative differences between the two (three) tested dynamics.

Compared to averaging over many unaligned runs, random event alignment offers a significant advantage. While the former method can—in a computationally feasible way—only incorporate short dynamical trajectories without succumbing to noise (electronic supplementary material, figure S7), our approach allows definitions of edge importance that are based on either long or short-time series. This can provide qualitatively different information, furthering our understanding of the system in a way that would not be possible without alignment (see our analysis of the macaque cortical area network as an example). Furthermore, we checked that under random event alignment, a meaningful range of the edge importance is obtained even after an extended simulation of SER dynamics (electronic supplementary material, figure S8). This implies that the removal of different edges has significantly different effects on long-term behaviour, and this distinction is not overshadowed by the divergence between different runs.

We discovered that the edge importance in the SER model is strongly correlated with centrality measures for both types of the network. Surprisingly, in the case of the macaque network IDC and IEC were weakly correlated, and the only relevant centrality measure was the BC. There also exists a mostly negative correlation with the other measures we presented in this study, yet it is substantially smaller. The parametric structure of these correlations does not expose substantial differences between ER and BA topologies. In the case of negative correlations, the macaque network stood out once again, showing substantial to strong negative correlation with TO and ICC. This again points to the conclusion, that this biological network has some dynamical properties that are very distinct, from both of the tested artificial models.

On the contrary, CA dynamics exhibit much lower topological SC-FC affinity than SER. Interestingly, it also exposes a large difference between the ER and BA topologies, at least from the perspective of the parametric correlation structure. Overall edge importance in BA seems to be much more related to centrality measures, than in ER. This effect is most prominent in the high κ regime and decreases substantially in chaotic regions. Interestingly, on the BA network in the chaotic regime, there are some moderately strong negative correlations, especially for IDA.

This points to the conclusion, that from the point of view of the edge importance CA is more decoupled from the network topology than the SER, at least from the point of view of presented classical measures.

These discrepancies suggest, that for each dynamical model, different aspects of the network topology are important. However, a precise explanation requires more research in a few different directions. Future studies should also use other, more refined and specialized, tools.

Another important point to consider is the effect that the diversity of initial conditions has on edge importance. While we assume starting states to be completely random, in real-world systems they typically exhibit some local correlation and are, to an extent, aligned with the structural features of the network. The impact that such an initial state bias might have in classification problems has been discussed in [46], and its effect on dynamical edge importance can be partially understood from our comparison between transient and asymptotic behaviour. An enhanced fraction of highly important edges in the case of transients is the main qualitative difference between the two. Since using completely random initial states in conjunction with short transients highlights the sensitivity of the network to external stimuli, we expect that aligning the states with the network structure from the start would drastically reduce the magnitude of this effect. However, further work is required to fully grasp the implications of introducing such biases, especially in real systems.

Given the qualitative differences between the two dynamical models in terms of edge importance, applying our methods to other types of dynamics is an important direction for future work. This is true for both other representatives of the same dynamical classes (excitable dynamics and pattern formation) and models that have little connection to the scope of this study. Pursuing this would greatly enhance our understanding of dynamical edge importance, and can potentially uncover new practical applications. Developing an analytical version of our approach is a challenging prospect even for specific models, because with the removal of an edge emergent dynamical phenomena on a network may change unpredictably. However, in cases where resulting dynamical changes are not collective in nature, one avenue for achieving this could be extending the analytical work in [36] towards edge importance.

The presented method can also be used to evaluate the importance of edges not present in the original network. This is achieved by considering the graph with an added edge in place of the graph with a removed one for the modified run and computing the distribution of such edge importance for all possible absent edges.

Over the course of this study, we have shown that dynamical edge importance strongly depends on the dynamical process in question and its parameters, network architecture and modelling choices, as well as the interplay of those factors. However, definitions obtained by using markedly different types of dynamics highlight different aspects of the network’s inner workings and can be independent across vast regions of this parameter space, which gives rise to potential applications. Apart from identifying network architectures that perform well for multiple dynamical tasks at once, one could also expand on the final observation made in §4 and such independent definitions to discern the true function of a given network. In addition, once a comprehensive, carefully curated set of dynamical models is obtained, this approach could pave the way to a new formal definition of network function that is deeply rooted in dynamics and can be verified in practice.

Summarizing, we have demonstrated, that assumptions of our general approach enable scrutinized investigation of the problem, generating results that reach the point in a very effective and comprehensive way. One has to bear in mind, that this is still an initial stage of a much larger enterprise, that will yield a much deeper understanding of the SC–FC relationship.

## Data Availability

Macaque cortical area network [14]. Supplementary material is available online [47].

## Appendix A. Edge importance computation algorithms

In this appendix, we give detailed descriptions of various algorithms used for computing dynamical edge importance. Please refer to §2.6 for a textual explanation of specific steps.

### A.1. SER edge importance

**Parameters:**

(i) simple graph G with n nodes and m edges;
(ii) set of initial states, M ;
(iii) number of time steps, N ;
(iv) length of the transient, $N_{0}$ ; and
(v) number of SER runs per state, k .

The definition of relevant nodes on step 2.2.3 depends on the choice of including or excluding the region around the affected edge. If this region is to be included, this is the full set of network nodes, and $n_{r}=n$ . Otherwise, $n_{r}$ is the number of nodes not adjacent to endpoints of e . On step 2.2.4, normalization happens with respect to $n_{r}$ , to avoid introducing an artificial degree-dependent bias.

## A.2. Chaotic Cellular automation edge importance

**Parameters:**

(i) simple graph G with n nodes and m edges;
(ii) set of initial states, M ;
(iii) number of time steps, N ; and
(iv) length of the transient, $N_{0}$ .

## A.3. Convergent Cellular automation edge importance

In the algorithm below, attractor is represented by a list of tuples of length n , containing a single such tuple in case of fixed point. $a_{\text{original}}[j]$ denotes j -th tuple in the list $a_{\text{original}}$ (zero-based indexing). Denote by $Hamming(l_{1},l_{2})$ Hamming distance between equally long lists of equal-size tuple.

**Parameters:**

(i) simple graph G with n nodes and m edges; and
(ii) set of initial states, M .
